# Tolerance and efficacy of off-label anti-interleukin-1 treatments in France: a nationwide survey

**DOI:** 10.1186/s13023-015-0228-7

**Published:** 2015-02-15

**Authors:** Linda Rossi-Semerano, Bruno Fautrel, Daniel Wendling, Eric Hachulla, Caroline Galeotti, Luca Semerano, Isabelle Touitou, Isabelle Koné-Paut, the MAIL1 (Maladies Auto-inflammatoires et Anti-IL-1) study Group on the behalf of CRI (Club Rhumatisme et Inflammation)

**Affiliations:** Department of Paediatric Rheumatology, Hôpital de Bicêtre, APHP, National Reference Centre for Auto-inflammatory Diseases, Le Kremlin-Bicêtre, University of Paris Sud, CHU de Bicêtre. 78 Rue du Général Leclerc, 94270 Le Kremlin, Bicêtre France; Department of Rheumatology, Hôpital La Pitié Salpêtrière, APHP; UPMC, GRC 08, Institut Pierre Louis d’Epidémiologie et Santé Publique, Paris, France; Department of Rheumatology, University Teaching Hospital, CHRU Besançon, and University of Franche-Comté, Besançon, France; Centre de Référence des Maladies Autoimmunes et systémiques Rares, Service de Médecine Interne, Hôpital Claude Huriez, Université Lille Nord-de-France, Lille Cedex, France; Department of Rheumatology, Hôpital Avicenne, APHP, INSERM UMR 1125, Paris 13 University, Bobigny, France; Maladies Auto-inflammatoires, Laboratoire de Génétique, Hôpital A de Villeneuve, CHRU Montpellier, Montpellier, France

**Keywords:** Auto-inflammatory diseases, IL-1, Anakinra, Canakinumab, Tolerance, Off-label

## Abstract

**Background:**

Despite their limited licensed indications, anti–interleukin-1 (anti–IL-1) agents are often used in clinical practice for an increasing number of auto-inflammatory diseases. We conducted a national cross-sectional observational study from January 2011 to January 2013 to record the off-label use of such agents in France. We aimed to estimate the off-label use of anti–IL-1 treatments in France, assess their efficacy in rare diseases, and increase the reporting of their possible side effects.

**Methods:**

Physicians answered a questionnaire that covered patient and disease data, anti–IL-1 agent use, efficacy and adverse events. The study involved adult or paediatric patient who had received an anti–IL-1 agent after January 2005 in France.

**Results:**

In total, 189 patients from 38 centres were included. The main diseases were adult-onset Still’s disease (AOSD) (35), gout (28), systemic juvenile idiopathic arthritis (27), cryopyrin-associated periodic syndrome (CAPS) (21), familial Mediterranean fever (14) and mevalonate kinase deficiency (12). The main off-label used agent was anakinra, used at least once for 185 patients, with canakinumab used for 25. Anakinra was effective in most patients (90%), with higher complete clinical response rates for Schnitzler’s syndrome, gout, CAPS and AOSD. Overall, 58% of patients showed at least one adverse event, mainly minor injection-site reactions. The main reported serious adverse event was severe infection. Injection-site reactions and liver toxicity were significantly more frequent in children than adults. The main non-cutaneous adverse event was liver toxicity, significantly associated with treatment duration. Weight gain was reported in about 10% of patients and was associated with treatment duration and CAPS. Canakinumab was rarely used and showed better cutaneous tolerance than anakinra but similar rates of non-cutaneous and severe adverse events.

**Conclusions:**

Anakinra was well tolerated and effective in most patients with various inflammatory diseases. The main adverse events were mild injection-site reactions, especially in children. The survey allowed for collecting limited information on the off-label use of canakinumab.

**Electronic supplementary material:**

The online version of this article (doi:10.1186/s13023-015-0228-7) contains supplementary material, which is available to authorized users.

## Background

The auto-inflammatory diseases (AIDs) cover a wide spectrum of systemic inflammatory diseases ranging from rare Mendelian to polygenic disorders whose pathogenesis lies in deregulation of interleukin-1 beta (IL-1β) secretion [1,2]. The diseases manifest as recurrent fevers, variously affecting other organs such as joints, skin, eyes, nervous system and serous tissues. Levels of acute-phase reactants are markedly elevated during flares and classically disappear (or are markedly reduced) in between flares. Other polygenic diseases such as adult-onset Still’s disease (AOSD), systemic juvenile idiopathic arthritis (sJIA), Beçhet’s disease and gout, also belong to the AIDs, in that deregulation of IL-1 is involved, at least in part, in their pathogenesis [3].

The use of anti–IL-1 treatments for these conditions is continually increasing, most often in an off-label setting and without standardized monitoring. Thus, we lack information on their medium- and long-term effectiveness and side effects.

At present, 4 molecules have been manufactured (Additional file 1: Table S1); 2 are available in France: anakinra, a short half-life IL-1 receptor antagonist (IL-1RA) approved for rheumatoid arthritis (RA) and since November 2013 for CAPS, and canakinumab, a long half-life monoclonal anti–IL-1β selective antibody, approved for CAPS and for sJIA and gouty arthritis since September and March 2013, respectively.

These drugs are given for a variety of rare diseases, which limits the feasibility of randomised controlled trials (RCTs). Therefore, we conducted a nationwide cross-sectional observational study to describe the indications for their off-label use and gather information about their tolerance and efficacy. The main objectives of the study were to 1) describe and analyse the efficacy and safety of anti–IL-1 agents in real-life clinical practice and 2) collect descriptive data on both adult and paediatric patients receiving anti–IL-1 agents in terms of the specific diseases that led to their off-label use.

## Methods

Physicians answered a questionnaire downloaded from the website of the national scientific society “Club Rhumatisme et Inflammation” (www.cri-net.com) that covered patient demographics, disease data, and anti–IL-1 treatment (type, dose, duration, effect on disease and adverse events (AEs). Eligible patients were adult and children who had received an off-label anti–IL-1 agent after January 2005. Physicians provided retrospective anonymous data from medical records, which were entered into a database.

### Collected information

Collected data included patient age, gender, disease, disease duration, clinical symptoms and signs, frequency of attacks, inflammatory biological markers (C-reactive protein [CRP] level before and after anti–IL-1 introduction). Other medications (disease-modifying anti-rheumatic drugs [DMARDs] and corticosteroids) before, during and after anti–IL-1 treatment were reported. Data on anti–IL-1 treatment included the type (name) of the drug, dose, frequency of injections, the date of disease onset, date and reason for discontinuation if discontinued, the effect on clinical symptoms and signs, and the occurrence of AEs.

### Assessments and definitions

Response to anti–IL-1 treatment was graded as complete, partial or failure. The evaluation was retrospective and not standardized, but we delineated several global response criteria, suitable for all diseases, including inflammatory marker normalisation (CRP level < 10 mg/L). Complete response or remission was retained when signs of active disease were absent and inflammatory markers had normalised. Partial response was retained when complete response was not achieved but clinical improvement was evident according to the treating physician.

Tolerance: the following AEs were specified in the questionnaire: pain and injection-site reactions (swelling, erythema, ecchymosis, pruritus), respiratory infections, neutropenia (≤1000/mmc), hepatic cytolysis (2-fold the normal value), other liver abnormalities, lipid profile abnormalities, and other freely reported AEs. Severe infections were researched and other severe AEs (SAEs) were freely reported. We also collected information on weight gain with anti–IL-1 treatment.

### Statistical analysis

Given the high variability in sample size for each disease, general statistics are reported as both mean ± SD and median ± interquartile range (IQR). All descriptive results are given with 95% confidence intervals (95% CIs). Categorical variables were compared by chi-square test with Yates continuity correction when required. Quantitative variables were compared by two-sided independent-samples *t* test. Significance level was set at p < 0.05. The association of s between patient-related variables and tolerance was studied by both univariate and multivariate analysis. For multivariate analysis, a stepwise logistic regression model included all explanatory variables showing univariate association with a p ≤ 0.2 with the dependent variables. Variables considered clinically relevant could be included despite the lack of univariate association. Odds ratios (ORs) are given with 95% CIs. For stratified explanatory variables, the chi-square test for trend was used to study the trend for positive association with dependent variables.

### Ethics

According to our local regulations, Institutional Review Board approval was not required for the study, but patients received detailed information on the study and were included only if they did not agree to electronic treatment of their data.

## Results

### Baseline patient characteristics

We included 189 patients (100 males), from 38 centres (29 adult centres and 9 paediatric rheumatology centres) (disease data in Table 1). At the time of anti–IL-1 introduction, 139 patients were adults, and 50 were children or adolescents (<18 years old). The mean age at treatment onset for children and adolescents was 8.3 ± 4.9 years (y), with median age 7.2 y (IQR: 12.5-3.5 = 9, total range (TR): 17.1-0.5 = 16.6). The mean age of adult patients was 46.6 ± 16.6 y, with median age 47.4 y (IQR: 57.3-33.0 = 24.3; TR: 86.3-18.6 = 67.7).

**Table 1:** **Baseline disease data**

**Disease**	**No. of patients**	**M/F**	**Median age** * **(y) (IQR, TR)**	**Median disease duration** * **(y) (IQR)**
**AOSD**	35	12/23	40.9 (22.4, 21.4-79.4)	4.4 (7.4, 0.04-46.9)
**Gout**	28	24/4	57.4 (11.5, 29.0-86.3)	1.6 (8.5, 0.03-37.2)
**SJIA**	27	17/10	7.3 (9.35, 2.1-29.1)	1.4 (5.2, 0.11-24.1)
**CAPS**	21	11/10	25.9 (22.5, 3.8-66.3)	20.7 (25.3, 0.5-54.7)
**FMF**	14	4/11	21.1 (33.7, 5.9-60.8)	13.1 (19.5, 5.3-42.9)
**MKD**	12	5/7	9.5 (14.9, 1.4-36.1)	9.5 (15.6, 0.83-34.9).
**SAPHO**	9	4/5	49.1 (18.8, 25.2-59.0)	10.6 (14, 1.2-26.3)
**Schnitzler’s syndrome**	7	5/2	55.3 (22.0, 49.9-76.2)	7.4 (6.2, 3.5-13.7)
**Spondyloarthritis**	5	4/1	44.1 (18.9, 31.2-72.5)	10.3 (7.3, 5.1-13.4)
**Vasculitis**	4	3/1	69.5 (18.6, 58.7-83.6)	6.7 (6.1, 3.8-15.9)
**Chondrocalcinosis**	4	1/3	67.9 (18.8, 46.8-83.6)	3.7 (2.8, 0.5-10.4)
**GPP**	3	2/1	55.5 (21.1, 44.3-72.4)	17.1 (13.4, 8.5-35.5)
**Polychondritis**	3	1/2	42.2 (27.5, 29.8-66.4)	9.1 (10.9, 8.3-30.1)
**TRAPS**	3	1/2	47.8 (29.5, 12.5-51.7)	31.2 (19.1, 9.3-47.5)

*At time of anti–IL-1 treatment onset.

M: male, F: female, AOSD: adult-onset Still’s disease, sJIA: systemic juvenile idiopathic arthritis, CAPS: cryopyrin-associated periodic syndrome, FMF: familial Mediterranean fever, MKD: mevalonate kinase deficiency, SAPHO: synovitis, acne, pustulosis, hyperostosis, osteitis, GPP: generalized pustular psoriasis, Vasculitis: giant cell arteritis (2) and polyarteritis nodosa (2), TRAPS: tumor necrosis factor receptor-associated periodic syndrome, IQR: interquartile range, TR: total range.

The diseases were AOSD (n = 35), gout (n = 28), sJIA (n = 27), anakinra-treated CAPS (n = 21), familial Mediterranean fever (FMF) (n = 14), mevalonate kinase deficiency (MKD) (n = 12); synovitis, acne, pustulosis, hyperostosis, osteitis (SAPHO) syndrome (n = 9); Schnitzler’s syndrome (n = 7); spondyloarthritis (n = 5); vasculitis (giant cell arteritis, n = 2; polyarteritis nodosa, n = 2); chondrocalcinosis (n = 4); generalized pustular psoriasis (GPP) (n = 3); tumor necrosis factor receptor-associated periodic syndrome (TRAPS) (n = 3); relapsing polychondritis (n = 3); NLRP12-asociated periodic syndrome (NAPS12) (n = 2); and other diagnoses (n = 12) (Table 1).

### Anti–IL-1 treatments

#### Anakinra

The main off-label anti–IL-1 agent used was anakinra, used at least once in 185 patients. Most treated patients received daily injections, which for a few patients in clinical remission could be spaced out. All adult patients received 100 mg/day, and children received a dose ranging from 1 to 6 mg/kg/day. Anakinra was administered as on-demand treatment (<15 days) in some patients with gout (n = 12), FMF (n = 3), chondrocalcinosis (n = 1) and neutrophilic dermatosis (n = 1).

Partial to complete efficacy was reported for 90.3% of patients (95% CI: 85.7 to 94.3) (Table 2). Complete clinical response rates varied, being higher in Schnitlzler’s syndrome (83%), gout (78%), CAPS (72%) and AOSD (54%). Overall, 40% of patients were still being treated at last visit (Table 2). Inefficacy or loss of efficacy in patients with AOSD and SJIA and persistent remission in patients with gout, were the principal reasons for anakinra withdrawal. The reasons for anakinra withdrawal and switch to canakinumab for each disease are reported in Table 3.

**Table 2:** **Anakinra efficacy by disease**

**Disease**	**No. of patients**	**Clinical response (available data)**			**Median treatment duration (days) (IQR)**	**Associated** * **treatment**	**CCS**	**DMARDs**	**Still treated**	**Associated treatment reduction**
		**None**	**Partial**	**Total**						
**AOSD**	35	3 (8.6%)	12 (34.3%)	19 (54.3%)	461 (1164)	28/34 (82.4%)	24/28 (85.7%)	26/28 (92.9%)	15/35 (42.9%)	25/34 (73.5%)
**Gout**	28	0	6 (21.4%)	22 (78.6%)	7 (96.5)	20/27 (74.1%)	7/20 (35%)	0	3/24 (12.5%)	22/24 (91.7%)
**sJIA**	26	3 (11.5%)	12 (46.2%)	11 (42.3%)	502 (1154)	22/23 (95.7%)	20/22 (90.1%)	9/22 (40.9%)	10/26 (38.5%)	22/25 (88%)
**CAPS**	21	0	6 (28.6%)	15 (72.4%)	1059 (1235)	7/18 (38.9%)	3/8	2/9	10/21 (47.6%)	11/13
**FMF**	13	1 (7.6%)	6 (46.2%)	6 (46.2%)	390 (814.5)	10/12 (83.3%)	4/12 (33.3%)	2/12 (16.7%)	7/13 (53.8)	6/9 (66.7%)
**MKD**	10	0	7 (70.0%)	3 (30.0%)	266 (448)	3/4	1/4	0/4	3/10	3/3
**SAPHO**	9	4 (44.4%)	4 (44.4%)	1 (11.1%)	86 (337)	4/8	1/4	1/4	2/9	3/6
**Schnitzler’s syndrome**	7	1 (16.7%)	0	5 (83.3%)	479 (1227)	4 (/5)	2/4	1/4	4/7	5/6
**Spondyloarthritis**	5	4	1	0	30 (10–91)	3/5	1/5	0/5	0/5	3/5
**Vasculitis**	4	0	2	1	424 (94–916)	3/3	3/3	1/3	2/4	3/3
**Chondro** **calcinosis**	4	1	2	1	52 (6–227)	2/4	1/4	1/4	2/4	2/4
**GPP**	3	0	0	3	56 (6–129)	2/3	1/3	0/3	1/3	2/3
**Polychondritis**	3	0	2	1	395 (110–1961)	3/3	3/3	3/3	1/3	2/3
**TRAPS**	3	0	2	1	215 (29–225)	3/3	3/3	0/3	2/3	2/3
**NAPS12**	2	0	2	0	797	2/2	0/2	0/2	0/2	0/2

CCS: corticosteroids. AOSD: adult onset Still’s disease, sJIA: systemic juvenile idiopathic arthritis, CAPS: cryopyrin associated periodic syndrome, FMF: familial Mediterranean fever, MKD: mevalonate kinase deficiency, Vasculitis: giant cell arteritis (2) and polyarteritis nodosa (2), GPP: generalized pustular psoriasis, TRAPS: tumor necrosis factor receptor-associated periodic syndrome, NAPS 12: NLRP12-associated periodic syndrome, IQR: interquartile range.

*Several patients received colchicine with anakinra: 13 Gout, 8 FMF, 2 NAPS 12, 1 AOSD and 1 sJIA.

**Table 3:** **Reasons for anakinra withdrawal**

**Disease**	**No. withdrawals/treated patients**	**Inefficacy or loss of efficacy**	**Adverse event**	**Persistent remission**	**On-demand treatment**	**Patient request**	**Switch to canakinumab**
**AOSD**	20/35	14	3	1	0	2	2
**Gout**	18/28	0	0	17	1	0	0
**SJIA**	16/26	7	3	5	0	1	6
**CAPS**	11/21	1	1	1	0	8	3
**MKD**	7/10	2	2	0	0	3	4
**FMF**	6/13	1	3	0	1	1	3
**SAPHO**	7/9	5	2	0	0	0	0
**Schnitzler’s syndrome**	3/7	1	2	0	0	0	1
**Spondylo arthritis**	5/5	2	3	0	0	0	0
**Vasculitis**	2/4	2	0	0	0	0	2
**Chondro calcinosis**	2/4	1	0	1	0	0	0
**TRAPS**	1/3	1	0	0	0	0	0
**GPP**	2/3	1	0	1	0	0	0
**Polychondritis**	1/3	1	0	0	0	0	0
**NAPS12**	2/2	2	0	0	0	0	0

AOSD: adult onset Still’s disease, sJIA: systemic juvenile idiopathic arthritis, CAPS: cryopyrin associated periodic syndrome, FMF: familial Mediterranean fever, MKD: mevalonate kinase deficiency, Vasculitis: giant cell arteritis (2) and polyarteritis nodosa (2), GPP: generalized pustular psoriasis, TRAPS: tumor necrosis factor receptor-associated periodic syndrome, NAPS 12: NLRP12-associated periodic syndrome.

#### Canakinumab

Canakinumab was used in 25 patients, mainly children (18 vs. 7 adults), as second-line treatment after anakinra for most (21). This medication was administered every 8 weeks for most AIDs except AOSD and sJIA, one patient with MKD and another with TRAPS who received canakinumab every 4 weeks. All adult patients and adolescents >40 kg received 150 mg; paediatric patients (<40 kg) received doses ranging from 2 to 7 mg/kg. Higher doses were administered for MKD and sJIA.

Global canakinumab efficacy was comparable to that of anakinra: 84% of patients (95% CI: 69.6 to 98.4) were clinical responders, and 42% (95% CI: 22.7 to 61.4) were complete responders (Table 4). Median treatment duration was 445 days (IQR: 126–749 = 623); 60% of patients were still receiving treatment at the last visit (Table 4). Five patients received canakinumab after anakinra failure: 1 achieved complete clinical response, and 4 were partial responders.

**Table 4:** **Canakinumab efficacy (used in 25 patients)**

**Disease**	**Patient no.**	**First anti–IL-1**	**Dose and frequency**	**Clinical response**	**Treatment duration (d)**	**Associated treatment**	**CCS**	**DMARDs**	**Still treated**
**AOSD**	1	No	150 mg/4 wk	No	148	No	No	No	No
**AoSD**	2	No	150 mg/8 wk	Total	31	Yes	Yes	No	Yes
**sJIA**	3	No	4 mg/kg/4 wk	Partial	61	Yes	Yes	Yes	No
**sJIA**	4	No	150 mg/8 wk	Total	499	UK	UK	UK	No
**sJIA**	5	No	150 mg/4 wk	No	31	Yes	Yes	Yes	No
**sJIA**	6	No	4 mg/kg/4 wk	Partial	28	Yes	Yes	No	No
**sJIA**	7	No	3 mg/Kg/4 wk	No	28	Yes	Yes	No	No
**sJIA**	8	No	4 mg/kg/4 wk	No	28	Yes	Yes	No	No
**sJIA**	9	**Yes**	4 mg/kg/4 wk	Partial	538	Yes	Yes	Yes	No
**FMF**	10	No	2 mg/kg/8 wk	Partial	731	Yes^#^	No	No	Yes
**FMF**	11	No	2 mg/kg/8 wk	Partial	1151	Yes^#^	No	No	Yes
**FMF**	12	No	2 mg/kg/8 wk	Total	589	Yes^#^	No	No	Yes
**FMF**	13	**Yes**	2 mg/Kg on demand	Total	966	Yes^#^	No	No	Yes
**MKD**	14	**Yes**	2 mg/kg/8 wk	Partial	802	Yes	Yes	No	Yes
**MKD**	15	**Yes**	2-3* mg/kg/8 wk	Partial	1112	No	No	No	Yes
**MKD**	16	No	3 mg/kg/8 wk	Total	1101	UK	UK	UK	Yes
**MKD**	17	No	2 mg/kg/8 wk	Total	957	No	No	No	Yes
**MKD**	18	No	7 mg/kg/8 wk	Partial	558	Yes**	No	No	Yes
**MKD**	19	No	3.5 mg/kg/8 wk	Total	163	UK	UK	UK	Yes
**Vasculitis**	20	No	150 mg/8 wk	Partial	181	Yes	Yes	No	No
**Vasculitis**	21	No	150 mg/8 wk	UK	UK	UK	UK	UK	No
**Schnitzler’s syndrome**	22	No	150 mg/8 wk	Partial	385	No	No	No	Yes
**TRAPS**	23	No	2 mg/kg/4 wk	Total	170	Yes***	No	No	Yes
**Erdheim Chester**	24	No	2 mg/kg/8 wk	Partial	679	Yes	Yes	No	Yes
**Blau syndrome**	25	No	150 mg/8 wk	Total	390	Yes	Yes	No	Yes

AOSD: adult onset Still’s disease; sJIA: systemic juvenile idiopathic arthritis; FMF: familial Mediterranean fever; MKD: mevalonate kinase deficiency, Vasculitis: giant cell arteritis (2) and polyarteritis nodosa (2), TRAPS: tumor necrosis factor receptor-associated periodic syndrome.

^#^FMF: all patients received colchicine with canakinumab.

*MKD patient no. 15: initial dose 2 mg/kg, then augmented to 3 mg/Kg.

*MKD patient no. 18: adalimumab was associated at the time of canakinumab onset.

***TRAPS: enalapril was given with canakinumab.

CCS: corticosteroids.

### Drug efficacy per disease

Overall data for drug efficacy are in Tables 2, 3 and Additional file 1: Table S2 (single cases of AIDs) for anakinra treatment and in Table 4 for canakinumab treatment.

### Tolerance of anti–IL-1 treatment

#### Anakinra

Overall, 58% of patients (95% CI: 50.3-65.3) who received anakinra experienced at least one AE. Most AEs were cutaneous: minor injection-site reactions (39% of patients) and injection-site pain (36%). Another frequent AE was liver enzymes elevation, which was reported in 14 patients (7%). Weight increase was reported as a side effect in 11% of patients.

AEs were significantly more frequent in paediatric than adult patients (90.2%, 95% CI: 77.5-96.1 vs 48.2%, 95% CI: 39.9-56.5, p < 0.0001) mainly because of a higher number of injection-site reactions in paediatric than adult patients (58.5%, 95% CI: 43.4-72.3 vs. 33.3%, 95% CI: 25.9-41.6, p < 0.01) and pain at the injection site (68.3%, 95% CI: 53.0-80.4 vs. 26.7%, 95% CI: 19.9-34.7, p < 0.0001). Liver toxicity was more frequent in children than adults (17%, 95% CI: 8.5-31.3 vs. 4.4%, 95% CI: 2.0-9.4, p < 0.05).

Only a few patients presented mild respiratory infections (2.8%), lipid abnormalities (1.7%) and neutropenia (1.1%). The frequency of these AEs did not differ between children and adults.

Overall, 9% of patients (CI: 5.2-13.6) presented an SAE, mainly severe infection (5.1%), with no difference in frequency between children and adults (12.2%, 95% CI: 5.3-25.6 and 7.4%, 95% CI: 4.0-13.1) (Table 5). Anakinra (2 mg/Kg/day, treatment duration 156 days) led to severe toxidermia in a child with sJIA with associated chronic myocarditis; the child died 3 days after anakinra withdrawal due to disease flare with acute myocarditis. An adult with Schnitzler’s syndrome received anakinra for about 3 years, with rapid and persistent complete clinical response. Anakinra treatment was stopped because of lower airway infection and the discovery at the same time of oesophageal cancer. The patient died 1 month later due to oesophageal cancer for which smoke and alcohol abuse were retained as the major risk factors.

**Table 5:** **Serious adverse events (SAEs) in adults (n = 11) and paediatric patients (n = 5) receiving anakinra**

**Adults**			**Children**		
**Patient no.**	**Disease**	**SAE**	**Patient no.**	**Disease**	**SAE**
1	Gout	Pulmonary abscess	1	sJIA	MAS and EBV infection
2	AOSD	Pneumonia	2	sJIA	MAS and hepatitis
3	AOSD	VZV infection	3	sJIA	MAS, severe lipodystrophy and leishmaniasis*
4	AOSD	MAS and infection	4	sJIA	Death, toxidermia**
5	Schnitzler’s syndrome	Cancer, death	5	sJIA	Scarlet fever
6	MKD	Severe bronchitis			
7	Vasculitis	Sinusitis			
8	Polychondritis	Cutaneous abscess and osteitis			
9	Pustular dermatosis	Septicemia			
10	FMF	Quincke oedema			
11	GPP	Severe cutaneous allergic reaction			

AOSD: adult onset Still’s disease, sJIA: systemic juvenile idiopathic arthritis, FMF: familial Mediterranean fever, MKD: mevalonate kinase deficiency. Vasculitis: polyarteritis nodosa. GPP: generalized pustular psoriasis.

MAS: macrophage activation syndrome. EBV: Epstein Barr virus.

*Leishmaniasis in child no. 3 was previously published [41].

**Child no. 4 had a severe sJIA with associated chronic myocarditis; severe toxidermia developed with anakinra treatment and he died 3 days after anakinra withdrawal due to disease flare and acute myocarditis.

#### Canakinumab

Overall, 52% (95% CI: 32.9-70.1) of patients receiving canakinumab presented at least 1 AE, mainly mild respiratory infection (17%); 9% had liver toxicity, and only 4% had injection-site reactions. Other reported AEs were eczema and mood disorders. Paediatric and adult patients did not differ in frequency and type of AEs.

Overall, 13% of patients presented at least 1 SAE (mainly severe infections; seizures in 1 patient). One death was reported in an adult with severe polyarteritis nodosa. The patient was first treated with anakinra started at 100 mg/day and increased at 200 mg/day after 6 months (overall treatment duration 424 days), then with canakinumab at 150 mg every 8 weeks for 2 months and every 4 weeks afterwards (overall treatment duration: 181 days). The patient died because of staphylococcal pneumonia 1 month after the last canakinumab injection.

#### Anakinra vs. canakinumab

Anakinra- and canakinumab-treated patients in the overall population (both children and adults) did not differ in AE or SAE frequency. Conversely, more children had an AE with anakinra (90.2%) versus canakinumab (58.8%) (p < 0.05). This difference was related to poor local tolerance to anakinra (pain at injection site: 68.3% vs. 6%, p < 0.0001; injection-site reaction 58.5% vs. 0%, p < 0.0001). The 2 populations did not differ in non-cutaneous AEs and SAEs.

### Risk factors for AEs on anakinra

In patients treated with anakinra, the odds for developing at least an AE were higher for paediatric patients (OR 5.1, 95% CI: 2.1 to 12.3 p < 0.001), particularly in those with sJIA (OR 6.5, 95% CI: 1.9 to 22.7). Conversely, gout disease and background DMARDs treatment were both associated with lower odds of AE (Table 6). On multivariable analysis, only paediatric age, gout disease and DMARD treatment remained associated with an AE (Table 3). Anti–IL-1 treatment duration, associated corticosteroids or NSAIDs, and other diseases apart from gout and sJIA were not associated with AEs. In children, different anakinra daily doses were not associated with AEs (data not shown). Daily doses did not vary in adults.

**Table 6:** **Association between patient variables and the occurrence of adverse events with anakinra treatment**

	**Any adverse event**				**Non cutaneous adverse event**			
**Patient variables**	**Univariate analysis**		**Multivariate analysis**		**Univariate analysis**		**Multivariate analysis**	
	**OR (95% CI)**	**p**	**OR (95% CI)**	**p**	**OR (95% CI)**	**p**	**OR (95% CI)**	**p**
Paediatric vs. adult	5.67 (2.37 to 13.60)	**0.0001**	3.27 (1.16 to 9.21)	**0.03**	1.83 (0.90 to 3.73)	0.10	-	n.s.
Treatment duration	2.12 (1.15 to 3.89)	**0.02**	-	n.s	2.61 (1.34 to 5.13)	**0.005**	3.37 (1.46 to 7.80)	**0.01**
**Background treatment:**								
Methotrexate	0.44 (0.21 to 0.95)	**0.03**	-	n.s.	0.61 (0.26 to 1.41)	0.25	-	n.s.
All DMARDs	0.47 (0.23 to 0.98)	**0.04**	0.27 (0.11 to 0.64)	**0.003**	0.72 (0.33 to 1.59)	0.42		
Corticosteroids	1.17 (0.57 to 2.38)	0.68			0.82 (0.38 to 1.75)	0.62		
NSAIDs	1.38 (0.58 to 3.29)	0.47			0.67 (0.26 to 1.74)	0.41		
**Disease:**								
AOSD	1.36 (0.72 to 2.57)	0.35	-	n.s.	0.91 (0.46 to 1.79)	0.78		
sJIA	6.53 (1.87 to 22.7)	**0.004**	-	n.s.	1.42 (0.58 to 3.48)	0.45		
Gout	0.06 (0.02 to 0.21)	**0.0001**	0.05 (0.001 to 0.24)	**0.0002**	0.07 (0.01 to 0.51)	**0.01**	-	n.s.
CAPS	1.37 (0.52 to 3.61)	0.53	-	n.s.	1.97 (0.77 to 5.10)	0.16	-	n.s.

OR: odds ratio. 95% CI: 95% confidence interval, DMARDs: disease-modifying anti-rheumatic drugs, NSAIDs : non-steroidal anti-inflammatory drugs, AOSD: adult onset Still’s disease, sJIA: systemic juvenile idiopathic arthritis, CAPS: cryopyrin associated periodic syndrome. n.s.: not significant.

Higher than the median treatment duration increased, while gout disease decreased, the risk of non-cutaneous AEs (Additional file 1: Table S3). On multivariable analysis, only the association with treatment duration persisted, and we found a significant trend for increased odds of overall non-cutaneous AEs with increased quartile of treatment duration (p = 0.05). Methotrexate or any DMARDs background treatment did not protect against non-cutaneous AEs. SJIA was associated with SAEs on univariate analysis. No variable was related to SAEs on multivariate analysis (Additional file 1: Table S4).

We assessed risk factors for the most frequent non-cutaneous AEs: liver abnormalities and severe infections. Because of the high frequency of weight gain, we also assessed risk factors for this event. Paediatric age and higher than the median treatment duration were associated with poor liver tolerance on both univariate and multivariate analyses (Additional file 1: Table S4). Anakinra dose and type of disease were not associated with liver tolerance. No variables were associated with risk of serious infections.

CAPS disease and higher than the median anakinra treatment duration were associated with increased risk of weight gain (Additional file 1: Table S4) on both univariate and multivariate analyses.

We studied in detail the trend for increased risk of weight gain and elevated liver enzyme levels with increasing quartile of disease duration. We found a significant trend for increased weight gain and liver toxicity with increased quartile of disease duration (Additional file 1: Table S5). Conversely, no trend was found between treatment duration and severe infection.

## Discussion

The results of the survey support the efficacy and safety of anakinra in everyday clinical practice for a large number of AIDs and show a different tolerance profile between adult and paediatric patients. Canakinumab was rarely used in our patient population. Its more convenient administration schedule (every 4 or 8 weeks) versus daily anakinra injections is especially suitable for diseases requiring lifelong treatment, such as sJIA and MKD.

At the onset of our data repository, anti–IL-1 drugs were not licensed for AIDs in France. Since 2005, the use of such drugs has been reported for various rare AIDs for which almost no clinical trials have allowed licensing for use. We aimed to assess the magnitude of these prescriptions and determine whether still-unknown side effects might not have been reported to the manufacturers or health authorities. In addition, we wanted to collect information on the potential effectiveness of anti–IL-1 agents for very rare disorders, for which the feasibility of RCTs is limited.

Despite the limitations of retrospective data collection, this report describes the largest series of AOSD patients receiving anakinra [4-6]. Most had some clinical response, with a considerable proportion of complete responses (54.3%). Although most patients were evaluated as responders, about a half had discontinued treatment at last follow-up visit, mainly due to lack or loss of efficacy. Patients with sJIA had similar rates of overall clinical response and treatment discontinuation for poor efficacy despite the numerically lower rates of complete response as compared with AOSD. Similarly, in a randomised placebo-controlled trial [7], most sJIA patients had a rapid and remarkable response to anakinra, but loss of efficacy was observed in most over time. In our study, anakinra was withdrawn because of persistent remission in 5 of 16 cases. Seven sJIA patients received canakinumab, in most cases as second-line treatment after anakinra, with some clinical response in 4. Our data show less effectiveness of canakinumab in sJIA than in the literature [8].

All our CAPS patients received anakinra. Despite very long disease durations (median 20.7 y), clinical response was reported in all 21 patients, with complete response in most (72%). These results are in agreement with those reported in other series [9-13].

Together with the series from Ozçakar et al. [14], this is the largest series of FMF patients receiving anakinra (13 patients). In FMF, and in most cases of Mendelian AIDs, the rates of clinical response were good, and anakinra helped in reducing or discontinuing associated medications, especially corticosteroids. This finding agrees with previously reported case series [15-19]. Moreover, anakinra was highly effective in Schnitzler’s syndrome, which agrees with the literature [20-23]. The reporting of anti–IL-1 treatment efficacy on inflammatory dermatosis is extremely limited [24-26]. In our study, anakinra was highly effective in 7 patients with GPP (3), pustular dermatosis (2), deficiency of the IL-36 receptor antagonist (1), and neutrophilic dermatosis (1).

IL-1 inhibition represents an alternative for patients with gout with failure of or intolerance to standard medications, because the main mechanism of crystal-induced inflammation is the activation of NLRP3 inflammasome and the consequent excessive production of IL-1β [27]. Canakinumab was recently approved by the European Medicines Agency for refractory gouty arthritis on the basis of results of 2 RCTs [28]. All patients included in our survey were responders to anakinra and almost 80% achieved clinical remission. The drug was discontinued because of persistent remission in most patients. The excellent response to anakinra in gouty arthritis was shown in both outpatient series [29], and in medically complex hospitalized patients [30].

The study provides some elements to assess the tolerance of anti–IL-1 agents, especially anakinra, within a non-randomized, non-selected population of patients with rare diseases, often with long disease duration. Moreover, we could compare the tolerance profile of these drugs between adults and children. Overall, anakinra was well tolerated, with risk of at least 1 AE developing higher for children than adults. Among paediatric patients, we did not find any association between anakinra dose and AE risk, but the number of children receiving high doses (>4 mg/kg) was probably too small to draw any conclusions.

Pain and skin reactions at the injection site were the main treatment-related side effects and were significantly more frequent in children than adults. An open-label study with anakinra for sJIA and AOSD showed a higher number of cutaneous AEs for sJIA than AOSD, but the sample size was too small to reach significance [31]. Cutaneous AEs, reported in all studies with variable frequencies (from 20% to 90%), are usually transient and improve after the first month of treatment [7,31,32]. In fact, drug dose or treatment duration was not associated with cutaneous AEs. Background treatment with DMARDs, mainly methotrexate, was associated with reduced risk of cutaneous AE on both univariate and multivariate analyses. To the best of our knowledge, this association had never been reported.

The main non-cutaneous AEs were liver abnormalities (reported apart from macrophage activation syndrome [MAS]). Weight gain was also reported in a number of patients. Weight gain can be considered a benefit for some patients, such as paediatric patients, but can be considered an AE, if excessive, in adult patients. Treatment duration was the best predictor of liver abnormalities and weight gain. The risk for both these events increased significantly with longer treatment, which supports the need for monitoring weight and liver tolerance with long-term anakinra treatment. Importantly, weight gain, which involved 1 in 10 patients in the survey, is not reported in the summary description for anakinra [33]. The association of anakinra treatment duration and liver toxicity was not demonstrated in a 3-year open-label follow-up of a large RCT of rheumatoid arthritis [34]. In our study, children were at increased risk of liver abnormalities, with no difference between children and adults in other non-cutaneous AEs or SAEs.

The most frequent SAEs were serious infection. We found no variable associated with SAEs or serious infection except in patients with sJIA but only on univariate analysis. The increased risk of SAE may be related to the intrinsic disease severity, and the association with other severe systemic disorders in this study might have been unnoticed because of low sample size. After serious infection, MAS was a frequently reported SAE. A strong association exists between sJIA and MAS, which may be occult in up to 50% of patients [35,36]. Hence, establishing the real association between the risk of MAS and anti–IL-1 treatment is difficult, especially in this AID. Moreover, some cases of MAS successfully treated with anakinra have been published [37].

Patients with gout seemed to be protected against AE development, except SAEs. Nevertheless, after adjustment for disease duration, the relationship was no longer significant on multivariate analysis. The short treatment duration and not the disease itself could explain the low risk. Conversely, CAPS patients were at increased risk of excessive weight gain with anakinra, and adjustment for disease duration did not eliminate the association on multivariate analysis. Therefore, the risk may not be entirely justified by treatment duration, and disease-related features may play a role [32,38,39].

The survey provides limited information on canakinumab and confirms the better cutaneous tolerance as compared with anakinra. The main AEs (upper and lower respiratory infections) are consistent with that reported in the summary description for the product [40].

The main limitation of the study is its retrospective cross-sectional design, which limits the assessment of causality. Moreover, physicians could report AEs that were not mentioned in the questionnaire. This situation may limit the reporting of mild AEs and increases the risk of unmeasured confounding factors. Moreover, the heterogeneity of the diseases, with the consequent lack of standardized definitions of clinical response and the relatively low number of patients per category of disease, limited the assessment of efficacy. For instance, we could not determine whether non-responders to anti–IL-1 treatment might have received an insufficient dose to achieve complete response. For patients receiving canakimumab, the sample size limits any pertinent analysis of efficacy.

## Conclusions

Our study supports the good efficacy and tolerance of anakinra for a variety of inflammatory diseases. The main AE events were mild injection-site reaction, more frequent in children than adults. Treatment duration was associated with poor liver tolerance and weight gain, which is relevant to better define the anakinra tolerance profile. The information on off-label use of canakinumab is limited and warrants further investigation.

## Additional file

Additional file 1: Table S1-S5.
**Table S1.** Characteristics and indications for anti–interleukin-1 (anti–IL-1) treatment. **Table S2.** Other auto-immune diseases treated with anakinra. **Table S3.** Association between patient variables and the occurrence of serious adverse events and weight gain on anakinra treatment. **Table S4.** Association between patient variables and the occurrence of liver toxicity and severe infections on anakinra treatment. **Table S5.** Increase in odds ratios for weight gain, liver toxicity and severe infections depending on anakinra treatment duration.

## Abbreviations

AE: adverse events
AIDs: auto-inflammatory diseases
AOSD: adult onset Still’s disease
CAPS: cryopyrin-associated periodic syndrome
CeRéMAI: French National Reference Centre for Auto-inflammatory Diseases
CI: confidence interval
CINCA: Chronic Infantile Neurological Cutaneous and Articular syndrome
CRI: Club rhumatisme et inflammation
CRP: C-reactive protein
DITRA: deficiency of the IL-36 receptor antagonist
DMARDs: disease-modifying anti-rheumatic drugs
EMA: European Medicines Agency
FCAS: Familial Cold Autoinflammatory syndrome
FMF: familial Mediterranean fever
GPP: generalized pustular psoriasis
IL-1: interleukin-1
IL-1RA: IL-1 receptor antagonist
IQR: interquartile range
MAS: macrophage activation syndrome
MKD: mevalonate kinase deficiency
MWS: Muckle-Wells syndrome
NAPS12: NLRP12-associated periodic syndrome
NSAIDs: non-steroidal anti-inflammatory drugs
RA: rheumatoid arthritis
SAEs: severe adverse events
SD: standard deviation
sJIA: systemic juvenile idiopathic arthritis
TR: total range
TRAPS: tumor necrosis factor receptor-associated periodic syndrome
