# Supplementary material for: Tolerance and efficacy of off-label anti-interleukin-1 treatments in France: a nationwide survey
Source: Orphanet J Rare Dis. 2015 Feb 15;10:19. doi: 10.1186/s13023-015-0228-7 (PMC4340831; doi:10.1186/s13023-015-0228-7)
Supplement: Additional file 1: Table S1-S5. — Table S1. Characteristics and indications for anti–interleukin-1 (anti–IL-1) treatment. Table S2. Other auto-immune diseases treated with anakinra. Table S3. Association between patient variables and the occurrence of serious adverse events and weight gain on anakinra treatment. Table S4. Association between patient variables and the occurrence of liver toxicity and severe infections on anakinra treatment. Table S5. Increase in odds ratios for weight gain, liver toxicity and severe infections depending on anakinra treatment duration. [file 13023_2015_228_MOESM1_ESM.docx]

Additional file 1: table S1-S5

Table S1. Characteristics and indications for anti–interleukin-1 (anti–IL-1) treatment

| **Agent** | **Anakinra**  **(KINERET ®)** | **Canakinumab**  **(ILARIS ®)** | **Rilonacept**  **(ARCALYST ®)** | **Gevokizumab**  **(XOMA 052)** |
| --- | --- | --- | --- | --- |
| **Properties** | Recombinant, non-glycosylated protein, analogue of human IL-1RA.  Inhibits IL-1α, IL-1ß and IL-1R1. | Fully human monoclonal antibody.  Inhibits IL-1 ß. | Chimerical protein of the extracellular domains of IL-1RI and IL-1RAcP.  Inhibits IL-1α and IL-1ß. | Fully human monoclonal antibody.  Inhibits IL-1 ß. |
| **Plasma half-life** | 4-6 h | 21-28 days | 67 h | 21-28 days |
| **Dosages^*^ in paediatric patients (<40 kg)** | 1-10 mg/kg/day | 2-4 mg/kg/ up to 300 mg/4 or 8 weeks^**^ | 2.2 mg/kg up to 160 mg/ week | NA |
| **Dosages^*^ in adult patients** | 100 mg/day | 150 mg/8 weeks | 160 mg/kg/week | NA |
| **Approval** | FDA, EMA: RA (since 2001)  FDA: severe forms of CAPS (since 2013)  EMA: all forms of CAPS (since 2013) | FDA, EMA, Japan: all types of CAPS, adults and children>2 years (since 2009)  FDA and EMA: gout (since 2013)  FDA and EMA: sJIA (since 2013^◊^) | FDA: FCAS and MWS in adults and children>12 y | No approval yet by FDA and EMA  (ongoing clinical trials on Behçets disease uveitis and non-infectious uveitis, pioderma gangrenosum, type 1 diabetes |
| **Costs** | 11680 €/y | CAPS: 72000 €/y  sJIA: 150 000 €/y | 360000 €/y | NA |

IL-1RA: interleukin-1 receptor antagonist; IL-1RI: interleukin-1 receptor component; IL1RAcP: interleukin-1 receptor accessory protein; NA: not yet approved; FDA: US Food and Drug Administration; EMA: European Medicines Agency; CAPS: cryopyrin-associated periodic syndrome; FCAS: Familial cold autoinflammatory syndrome; MWS: Muckle-Wells syndrome; sJIA: systemic juvenile idiopathic arthritis. *Dosages according to RCP. ** every 4 weeks for sJIA, every 8 weeks for CAPS . ^◊^Not yet available for this indication in France.

Table S2. Other auto-immune diseases treated with anakinra.

| **Disease** | **Age (y)** | **Disease duration**  **(y)** | **Dose**  **(mg/d or mg/kg/d)** | **Clinical**  **response** | **Treatment duration**  **(d)** | **Associated**  **treatment** | **CCS** | **DMARDs** | **Still**  **treated** | **Reason for withdrawal** | **Associated**  **Treatment**  **reduction** | **Tolerance** |
| --- | --- | --- | --- | --- | --- | --- | --- | --- | --- | --- | --- | --- |
| **Multicentric**  **Castleman** | 14.0 | 7.3 | 1/kg/d | Partial | 803 | Yes | Yes | No | No | Loss of efficacy | Yes | No AE |
| **DITRA** | 0.5 | 0.5 | 2-5/Kg/d | Partial | 47 | Yes^1^ | Yes^1^ | No | Yes |  | Yes | Pain at injections site |
| **Erdheim Chester^2^** | 12.7 | 5.9 | 1/kg/d | Partial | 361 | Yes | Yes | No | No | Patient request | Yes | No AE |
| **Blau syndrome** | 26.7 | 25.9 | 100/d | Partial | 533 | Yes | Yes | No | No | Loss of efficacy | Yes | Pain and ISR^3^ |
| **Psoriatic arthritis** | 27.1 | 10.9 | 100/d | No | 114 | Yes | No | No | Yes | Inefficacy | Yes | No AE |
| **Pustular dermatosis** | 31.4 | 0.95 | 100/d | Partial | 22 | Yes^4^ | No | No | No | Loss of efficacy and AE | Yes | Serious  infection |
| **Neutrophilic**  **dermatosis** | 62.5 | UK | 100/d | Complete | OD | No | No | No | Yes |  | NA | Pain and ISR^3^ |
| **Hydrosadenitis**  **suppurativa** | 49.2 | 13.9 | 100/d | Partial | 578 | Yes^5^ | No | No | Yes |  | Yes | ISR^3^ |
| **Digital osteoarthritis** | 67.2 | 22.0 | 100/d | Partial | 22 | No | No | No | No | AE | NA | ISR worsening asthma |
| **Non-classified** | 34.5 | 1.4 | 100/d | Complete | 273 | Yes | Yes | No | Yes |  | Yes | Severe  neutropenia |
| **Non-classified** | 43.7 | 2.5 | 100/d | Partial | 366 | Yes | No | Yes | No | Loss of efficacy | No | No AE |
| **Non-classified** | 15.9 | 2.5 | 100/d | Partial | UK | Yes | Yes | No | No | Loss of efficacy and AE | UK | Pain and ISR^3^, generalized  itch |

CCS: corticosteroids. DITRA: Interleukin-36 receptor antagonist deficiency. UK: unknown. OD: on demand. ^1^Opioids and topic corticosteroids as associated treatment; ^2^The patient received canakinumab as second-line treatment (2 mg/kg every 8 weeks) with partial efficacy and mild adverse event (AE; respiratory infection); ^3^ISR : injection-site reaction; ^4^Acitretin as associated treatment ; ^5^Opioids as associated treatment.

Table S3. Association between patient variables and the occurrence of serious adverse events and weight gain on anakinra treatment

|  |  | Serious adverse event | | | | |  | Weight gain | | | | | | |
| --- | --- | --- | --- | --- | --- | --- | --- | --- | --- | --- | --- | --- | --- | --- |
| Explanatory Variable |  | Univariate Analysis | |  | Multivariate Analysis | |  | | Univariate Analysis | | |  | Multivariate Analysis | |
|  |  | OR (95% CI) | p |  | OR (95% CI) | p |  | | OR (95% CI) | | p |  | OR (95% CI) | p |
| Paediatric vs. adult |  | 1.56 (0.50 to 4.84) | 0.45 |  |  |  |  | | 1.88 (0.69 to 5.13) | | 0.25 |  | - | n.s. |
| Treatment duration |  | 1.89 (0.61 to 5.88) | 0.26 |  | - | n.s. |  | | 4.31 (1.38 to 13.60) | | **0.001** |  | 3.49 (1.10 to 11.22) | **0.04** |
| **Background treatment:** |  |  |  |  |  |  |  | | |  |  |  |  |  |
| Methotrexate |  | 0.40 (0.08 to 1.92) | 0.21 |  | - | n.s. |  | | 1.34 (0.45 to 3.97) | | 0.60 |  |  |  |
| All DMARDs |  | 0.53 (0.14 to 2.10) | 0.35 |  |  |  |  | | 1.37 (0.47 to 3.94) | | 0.57 |  |  |  |
| Corticosteroids |  | 1.25 (0.36 to 4.39) | 0.73 |  |  |  |  | | 0.57 (0.20 to 1.63) | | 0.29 |  | - | n.s. |
| NSAIDs |  | 0.68 (0.14 to 3.23) | 0.63 |  |  |  |  | | 1.20 (0.36 to 4.10) | | 0.76 |  |  |  |
| **Disease**: |  |  |  |  |  |  |  | |  | |  |  |  |  |
| AOSD |  | 2.26 (0.88 to 7.42) | 0.09 |  | - | n.s. |  | | 0.94 (0.34 to 2.61) | | 0.90 |  |  |  |
| sJIA |  | 3.63 (1.12 to 11.70) | **0.04** |  | - | n.s. |  | | 1.77 (0.54 to 5.87) | | 0.35 |  |  |  |
| Gout |  | 0.39 (0.05 to 3.13) | 0.32 |  |  |  |  | | 0.00 | | 0.99 |  |  |  |
| CAPS |  | 0.00 | 0.99 |  | - | n.s. |  | | 4.78 (1.57 to 14.53) | | 0.01 |  | 3.44 (1.10 to 10.74) | **0.02** |

OR: odds ratio, 95% CI: 95% confidence interval, DMARDs: disease-modifying anti-rheumatic drugs, NSAIDs: non-steroidal anti-inflammatory drugs, AoSD: adult-onset Still’s disease, sJIA: systemic juvenile idiopathic arthritis, CAPS: cryopyrin-associated periodic syndrome.

n.s.: not significant

Table S4. Association between patient variables and the occurrence of liver toxicity and severe infections on anakinra treatment

|  |  | Liver toxicity | | | | |  | Severe Infection | | | | | | |
| --- | --- | --- | --- | --- | --- | --- | --- | --- | --- | --- | --- | --- | --- | --- |
| Explanatory Variable |  | Univariate Analysis | |  | Multivariate Analysis | |  | | Univariate Analysis | | |  | Multivariate Analysis | |
|  |  | OR (95% CI) | p |  | OR (95% CI) | p |  | | OR (95% CI) | | p |  | OR (95% CI) | p |
| Paediatric vs. adult |  | 3.96(1.26 to 12.50) | **0.02** |  | 4.20 (1.11 to 15.91) | **0.04** |  | | 1.54 (0.36 to 6.41) | | 0.56 |  |  |  |
| Treatment duration |  | 3.63 (0.96 to 13.68) | 0.06 |  | 10.10 (1.16 to 87.30) | **0.04** |  | | 3.14 (0.62 to 16.03) | | 0.17 |  | - | n.s. |
| Background treatment: |  |  |  |  |  |  |  | | |  |  |  |  |  |
| Methotrexate |  | 0.22 (0.02 to 1.79) | 0.16 |  | - | n.s. |  | | 0.29 (0.03 to 2.43) | | 0.19 |  | - | n.s. |
| All DMARDs |  | 0.17 (0.02 to 1.39) | 0.10 |  | - | n.s. |  | | 0.54 (0.10 to 2.81) | | 0.47 |  |  |  |
| Corticosteroids |  | 0.91 (0.24 to 3.41) | 0.89 |  |  |  |  | | 1.02 (0.23 to 4.48) | | 0.97 |  |  |  |
| NSAIDs |  | 2.58 (0.67 to 9.88) | 0.17 |  | - | n.s. |  | | 1.19 (0.23 to 6.26) | | 0.83 |  |  |  |
| Disease: |  |  |  |  |  |  |  | |  | |  |  |  |  |
| AOSD |  | 1.84 (0.59 to 5.73) | 0.29 |  |  |  |  | | 1.69 (0.44 to 6.53) | | 0.45 |  |  |  |
| sJIA |  | 3.10 (0.87 to 10.93) | 0.08 |  | - | n.s. |  | | 3.38 (0.80 to 14.53) | | 0.10 |  | - | n.s. |
| Gout |  | 0.00 | 0.99 |  |  |  |  | | 0.76 (0.10 to 6.45) | | 0.80 |  |  |  |
| CAPS |  | 1.48 (0.30 to 7.24) | 0.63 |  |  |  |  | | 0.00 | | 0.99 |  |  |  |

DMARDs: disease-modifying anti-rheumatic drugs, NSAIDs: non-steroidal anti-inflammatory drugs, AOSD: adult-onset Still’s disease, sJIA: systemic juvenile idiopathic arthritis, CAPS: cryopyrin-associated periodic syndrome.

n.s.: not significant

Table S5. Increase in odds ratios for weight gain, liver toxicity and severe infections depending on anakinra treatment duration

|  | Weight gain | | | | Liver toxicity | | | | Severe infection | | | |
| --- | --- | --- | --- | --- | --- | --- | --- | --- | --- | --- | --- | --- |
| **Treatment Duration** | Result | | Odds Ratio (95%CI) | P for trend | Result | | Odds Ratio (95%CI) | p for trend | Result | | Odds Ratio (95%CI) | p for trend |
|  | Pos | Neg |  |  | Pos | Neg |  |  | Pos | Neg |  |  |
| First quartile | 1 | 36 | 1.00 (reference) | 0.01 | 1 | 37 | 1.00 (reference) | 0.04 | 1 | 37 | 1.00 (reference) | 0.23 |
| Second quartile | 3 | 36 | 3.00 (0.31 to 30.22) |  | 2 | 37 | 2.02 (0.18 to 23.02) |  | 1 | 37 | 1.00 (0.06 to 16. 9) |  |
| Third quartile | 6 | 38 | 5.68 (0.65 to 49.56) |  | 5 | 39 | 4.74 (0.54 to 42.54) |  | 3 | 39 | 2.84 (0.28 to 28.60) |  |
| Fourth quartile | 9 | 38 | 8.53 (1.03 to 70.73) |  | 6 | 38 | 5.90 (0.67 to 50.09) |  | 3 | 38 | 2.92 (0.29 to 29.73) |  |

Trend analysis of the association between quartiles of anakinra duration and the risk for weight gain, liver toxicity and severe infection. The ORs are referred to the first quartile of treatment duration.
